# Inequalities in regional excess mortality and life expectancy during the COVID-19 pandemic in Europe

**DOI:** 10.1038/s41598-024-54366-5

**Published:** 2024-02-15

**Authors:** Tamás Hajdu, Judit Krekó, Csaba G. Tóth

**Affiliations:** 1 HUN-REN Centre for Economic and Regional Studies, Budapest, Hungary; 2 HUN-REN Centre for Economic and Regional Studies, Hungary and Budapest Institute for Policy Analysis, Budapest, Hungary; 3 HUN-REN Centre for Economic and Regional Studies, Hungary and Corvinus University of Budapest, Budapest, Hungary

**Keywords:** Health care economics, Health policy, Public health, Disease prevention

## Abstract

Using data for 201 regions (NUTS 2) in Europe, we examine the mortality burden of the COVID-19 pandemic and how the mortality inequalities between regions changed between 2020 and 2022. We show that over the three years of the pandemic, not only did the level of excess mortality rate change considerably, but also its geographical distribution. Focusing on life expectancy as a summary measure of mortality conditions, we find that the variance of regional life expectancy increased sharply in 2021 but returned to the pre-pandemic level in 2022. The 2021 increase was due to a much higher-than-average excess mortality in regions with lower pre-pandemic life expectancy. While the life expectancy inequality has returned to its pre-pandemic level in 2022, the observed life expectancy in almost all regions is far below that expected without the pandemic.

## Introduction

In recent years, many empirical studies have demonstrated the significant mortality burden of the COVID-19 pandemic and its impact on life expectancy, using metrics such as excess deaths, reductions in life expectancy, or loss of years of life. Many of these papers have taken the country-level approach to expose the mortality burden of a single country^1–4^, to compare a group of countries^5–10^ or to analyze global trends of the COVID-19 pandemic^11,12^. Additionally, several country-specific case studies explored variations in the mortality burden within countries^13–16^, revealing significant differences across subnational regions. This highlights the need to shift towards geographic disaggregation to gain a deeper understanding of the factors influencing the mortality burden of the pandemic. However, to the best of our knowledge, only a few studies^17–20^ have been published so far that have investigated COVID-19 mortality at subnational level across multiple countries, focusing on the magnitude of regional differences in the mortality burden of the pandemic. Three studies focus on European regions or communities^17,19,20^ and a meta-analysis covers 74 countries from all continents, providing intra-country regional estimations for some of the countries^18^. Our first contribution to this literature is to extend the geographical coverage and the survey period, that enables us to provide a deeper insight into the changes in regional differences in mortality over successive phases of COVID-19.

In this paper, we comprehensively analyze the time trends in excess mortality rates and life expectancy at birth in Europe and their differences between NUTS 2 regions. Eurostat’s timely provision of weekly mortality data for European countries and subnational regions enabled us to examine the trends over three full years of the COVID-19 pandemic, from the beginning of 2020 to the end of 2022. In addition, using regional data allowed us to look more closely at geographical differences. We not only present changing trends in regional excess mortality over 2020–2022 and the pandemic's impact on diverting life expectancy from its long-term trend but also show how these mortality burden of the COVID-19 is related to the health capital of the population, measured by pre-pandemic life expectancy, of the European regions. Thus, we demonstrate how regional inequality in life expectancy at birth has changed in the years of COVID-19. Our focus extends beyond excess mortality rates to include a detailed examination of life expectancy since it is a commonly used summary measure of the mortality conditions of a population for a specific period unaffected by the population’s age structure. We also study the differences between observed and predicted trends in life expectancy across European regions.

Our paper also contributes to the literature addressing unequal death burden of COVID-19 along different socioeconomic indicators. Papers analyzing subpopulations within one country identified significant disparities: there is evidence that losses varied greatly by race and ethnicity in the US^2,21–23^. A meta-review encompassing 95 studies worldwide, examining geographical inequalities within countries, concluded that the vast majority of the studies revealed higher COVID-19 mortality rates in areas characterized by socioeconomic disadvantage compared to affluent areas^24^. We add to this literature by examining the role of the population’s health capital in the differences in excess mortality and change in life expectancy of the European regions. We consider health capital as something “that produces an output of healthy time”^25^ and might make individuals more resilient to various health shocks. In the empirical analysis, we measured it by the average life expectancy in the pre-pandemic years (2015–2019) since it adequately reflects the differences in the overall health status of the European regions. Life expectancy is a composite indicator, which is also correlated with various national level and regional level social and economic development factors, such as access to and the quality of the healthcare system, education and income levels and the population’s lifestyle and diet^26–29^. Therefore, in a broader sense, analyzing the impact of the pandemic by pre-pandemic life expectancy level can shed light on how advantaged and disadvantaged regions in terms of general health prospects coped with the burden of the pandemic.

We provide evidence of a potential feedback loop between life expectancy and excess mortality during the COVID-19 pandemic. To demonstrate this reciprocity, we use the life expectancy indicator in two ways. First, we apply pre-pandemic life expectancy (the average over 2015–2019) to measure health capital and investigate its association with the mortality burden of the pandemic. Second, we use the change in life expectancy during the pandemic as an indicator of the mortality impact caused by COVID-19. Additionally, we examine the fluctuations in the variance of life expectancy to demonstrate changes in regional disparities in mortality.

Focusing on NUTS 2 regions instead of countries has additional advantages: we obtain a more precise picture of the mortality situation, and the larger database provides more robust estimations. Since regional-level data on registered COVID-19 mortality is incomplete, often incomparable due to methodological differences, and published with a long-time lag, we do not examine COVID-19 deaths. Instead, we calculate regional-level excess mortality for European countries.

The novelty of our work is threefold. We present regional-level excess mortality for Europe to measure the mortality burden of COVID-19 for the entire period from the pandemic outbreak to the end of 2022. In addition, we analyze the regional association between health capital and COVID-19 excess mortality. Moreover, we reveal the changing inequalities in regional mortality by investigating the variance in life expectancy from 2019 to 2022.

In the next section, we introduce the databases and present the methods used in this article. We describe the estimation method for excess mortality, the calculation of annual life expectancy from age- and sex-specific mortality data, and the parameters of the regressions. In the next chapter, we present the excess mortality estimates for the whole period and the different years. Then we analyze the relationship between pre-pandemic life expectancy and regional excess mortality in the different subperiods. In the final section of the results, we show the change in the variance of annual life expectancy across Europe. We place our results in context in the Discussion part of the article. In the final section, we provide our conclusions.

## Data and methods

Our empirical analysis was based on Eurostat’s two publicly available datasets. The first is the number of weekly deaths by sex, five-year age group (from 0–4 to 90 years and older), and NUTS 2 region. The second is the population size by age, sex, and NUTS 2 region on January 1 each year. We used data for the years 2015–2022 and restricted the analysis sample to the countries of the European Union and the European Free Trade Association. Three countries were excluded: (i) Germany due to lack of age-specific mortality (five-year age groups), (ii) Ireland due to lack of mortality data for 2015–2019, and (iii) Norway due to significant changes in the geographical definition of the NUTS 2 regions in 2021 since no historical data were available for the new regions. In addition, five French overseas regions were excluded from the sample (Guadeloupe, Martinique, Guyane, La Réunion, and Mayotte). The final dataset comprised 201 NUTS 2 regions in 28 European countries (some smaller countries comprised only one region).

In Eurostat’s weekly mortality database, calendar weeks are defined according to the ISO 8601 standard. In this system, each year has 52 or 53 full weeks. The first week always includes January 4, but it can begin as early as December 29 of the previous year or as late as January 4. For our analysis, we restructured these data so that each day of the first and last week of the year belongs to the same year. The original weekly death counts are first distributed across the days of the given week (assuming that each day has the same number of deaths). Then, a new weekly database is created in which each year is divided into precisely 52 weeks, and the first calendar week contains the first seven days of the year (from January 1 to 7). This approach also means that the 52nd calendar week is 8 days long (except in leap years, when it is nine days).

To determine the excess mortality rate, we first calculated weekly mortality rates (deaths per million population) for each region-by-sex-by-age group using the weekly death counts and population on January 1. Then, we estimated predicted mortality rates from weekly mortality rates between 2015 and 2019. Specifically, in the 2015–2019 data, we estimated the following equation with ordinary least squares (OLS) regression:

1 $${M}_{rasyw}^{O}={\alpha }_{rasw}+{\beta }_{ras}\times {t}_{yw}+{\varepsilon }_{rasyw}$$

where M^O^ is the observed mortality rate (deaths per million population) in region *r*, age group *a*, sex *s*, year *y*, and calendar week *w*. Region-by-age-by-sex-by-week fixed effects (α) control for time-invariant differences between region-by-age-by-sex-by-week groups. These fixed effects effectively capture spatial, age- and sex-specific differences and the seasonality of the mortality rates. *t* is a discrete variable denoting time (year–week), so Eq. (1) also includes region-by-age-by-sex-specific linear time trends that control for gradual changes in mortality rates of the region-by-age-by-sex groups. ε is the error term. It should be noted that this specification led to identical results to an estimation where we ran regressions with calendar week fixed effects and a linear time trend separately for each region-age-sex cell. Similar fixed effects methods have been used in other studies to estimate excess mortality due to COVID-19^30–32^.

From Eq. (1), the predicted mortality rate (M^P^) can be obtained for all weeks in the analysis period (2015–2022) as:

2 $${M}_{rasyw}^{P}={\alpha }_{rasw}+{\beta }_{ras}\times {t}_{yw}$$

Next, we calculated excess mortality rates (M^E^) by subtracting the predicted mortality rates from the observed mortality rates:

3 $${M}_{rasyw}^{E}={M}_{rasyw}^{O}-{M}_{rasyw}^{P}$$

In the main analysis, we used the annual excess and predicted mortality rates for the total population. These were calculated as the weighted average of the age-specific (annual) excess and predicted mortality rates, where the weights are the population shares of the age groups on January 1:

4 $${M}_{rsy}^{E}=\sum_{a}\frac{{N}_{rasy}\sum_{w}{M}_{rasyw}^{E}}{{N}_{rsy}}$$

5 $${M}_{rsy}^{P}=\sum_{a}\frac{{N}_{rasy}\sum_{w}{M}_{rasyw}^{P}}{{N}_{rsy}}$$

where *a* runs from the 0–4 age group to the 90 + age group, *N*_rasy_ is the number of individuals in region *r* and age group *a* with sex *s* on January 1 of year *y,* and *N*_rsy_ is the total population in region *r* with sex *s* on January 1 of year *y*.

Region-specific life expectancy values (at birth) were obtained by first calculating the annual death counts for the 5-year age groups (the highest age group is 90 years and older) and combined with population figures for January 1 of the year in question. Next, life expectancy was calculated following the methods described by Chiang^33^.

While the standard methods use the mid-year population to calculate mortality rates and life expectancy, regional population data were only available until January 1, 2022; consequently, the mid-year population for 2022 was unknown. Due to this data limitation, the population at the beginning of the year was used for the calculations. However, it is shown in the Results section that this does not affect the study’s conclusions.

We also calculated predicted life expectancy based on the predicted death counts determined from the predicted mortality rates. These values show the predicted life expectancy that would have occurred if mortality had followed the time trend and seasonality of the years between 2015 and 2019. Our main analysis does not focus on sex-specific excess mortality rates and life expectancy but looks at the population as a whole. However, we also present some of our main results separately for males and females.

To examine how regional excess mortality rates are related to the pre-pandemic life expectancy (health capital), we used the average life expectancy for 2015–2019. These relationships were estimated using OLS regressions. In the baseline specification, since we aimed to demonstrate the correlation instead of recovering the causal impact of life expectancy, we estimated the relationship between excess mortality rates and pre-pandemic life expectancy without control variables. We also estimated a model including country fixed effects.

We also used the pre-pandemic life expectancy to define the top 20%, bottom 20%, and middle 60% of the regions. We analyzed the excess mortality rates and life expectancy trends of these three groups with different health capital levels and the time trend in the difference between the top and bottom 20%.

Bootstrapping technique was used to derive 95% confidence intervals for predicted and excess mortality rates, group-specific life expectancy, and variance of life expectancy.

## Results

**Figure 1:**
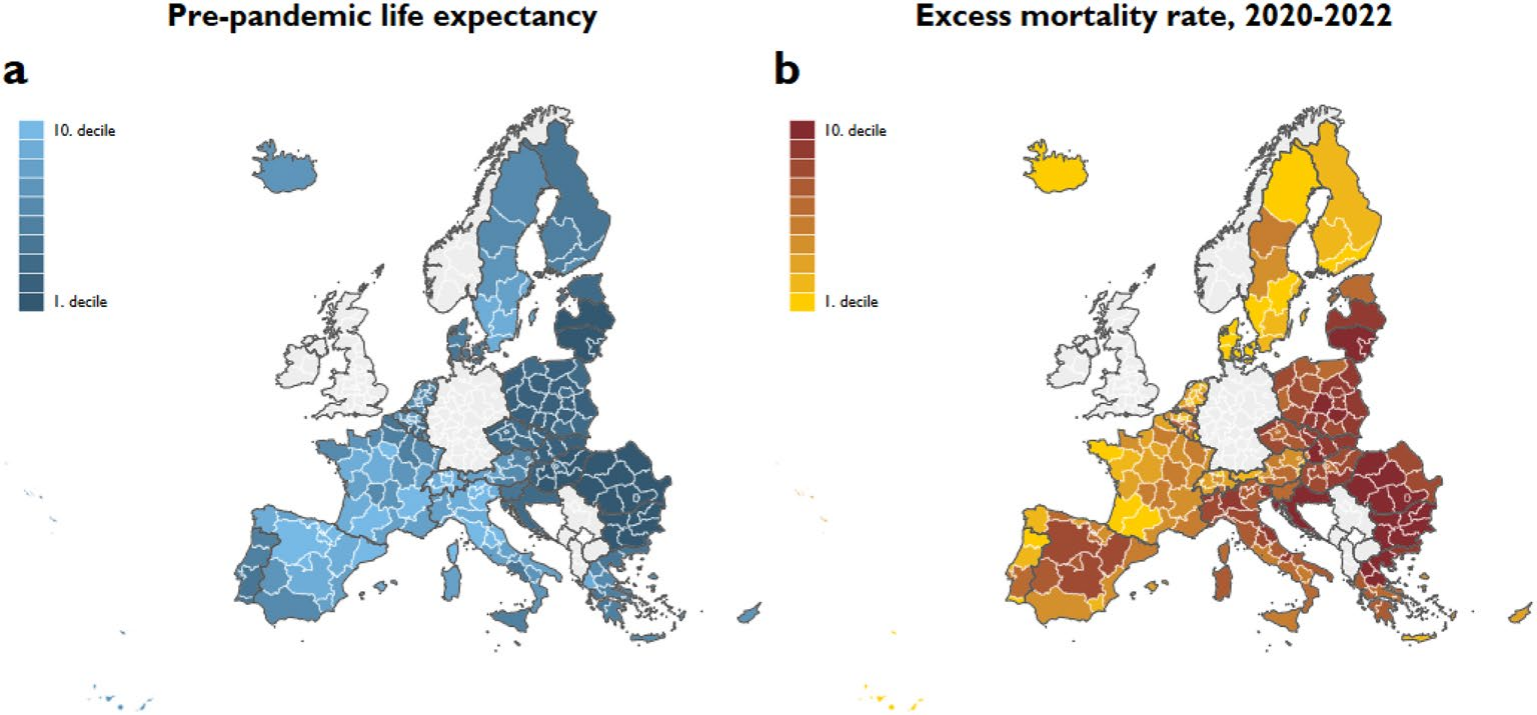
Excess mortality and pre-pandemic life expectancy. Notes: (**a**) Excess mortality rates reflect the difference between the observed and predicted mortality rates. Predicted mortality rates are projected from the observed mortality rates between 2015 and 2019. The projection accounts for seasonality and linear time trends in mortality rates. The excess mortality of the total population is the weighted average of the age-specific excess mortality rates, where the weights are the population shares of the age groups on January 1. (**b**) Pre-pandemic life expectancy is defined as the average life expectancy over 2015–2019.

Pre-pandemic life expectancy varied significantly between NUTS 2 regions in Europe, ranging from 73.6 to 84.7. Figure 1a highlights an East–West divide, with regions in France, Spain, Italy, and Switzerland falling into the highest decile and those in Bulgaria, Romania, Latvia, Lithuania, and Hungary the lowest decile of pre-pandemic life expectancy. The death toll from the COVID-19 pandemic also showed significant regional variation. The estimated excess mortality rate for the 2020–2022 period was lowest in Luxembourg (− 20 per million persons, 95% CI =  − 939 to 884) and highest in Northwestern Bulgaria (12,593 per million persons, 95% CI = 10,522 to 14,137) Most regions with excess mortality rates in the top deciles were in Central and Eastern Europe. However, extremely high excess mortality rates were also observed in some Mediterranean regions (Fig. 1b).

Variation between countries accounted for 91% of the total variance in regional pre-pandemic life expectancy, indicating that country-level institutional, environmental, and behavioral factors greatly affect the expected lifespan of the population. Nevertheless, intra-country variation was also remarkable. Similarly, the variation in the regional excess mortality rate for the 2020–2022 period was mainly due to national factors, explaining 80% of the overall variation in excess mortality per million population.

**Figure 2:**
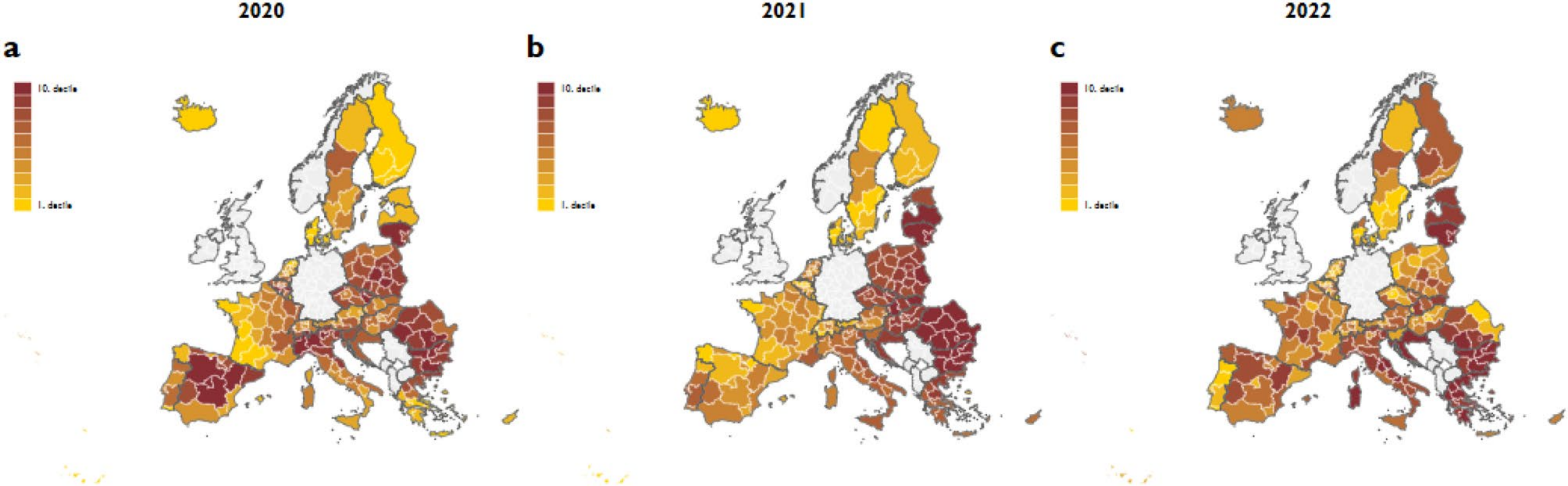
Excess mortality rate deciles by year. Notes: Excess mortality rates reflect the difference between the observed and predicted mortality rates. Predicted mortality rates are projected from the observed mortality rates between 2015 and 2019. The projection accounts for seasonality and linear time trends in mortality rates. The excess mortality of the total population is the weighted average of the age-specific excess mortality rates, where the weights are the population shares of the age groups on January 1.

The regional pattern in excess mortality rate changed considerably throughout the different phases of the pandemic (Fig. 2). The East–West divide was observed only in 2021, a year which accounts for 41% of total excess deaths between 2020 and 2022 in our sample of 201 European NUTS 2 regions. In 2020, high excess mortality rates were observed in several high-income regions in addition to some eastern regions. In 2022, the spatial pattern in excess mortality rate was less clustered, although many regions in Greece, Bulgaria, and the Baltic states had high excess mortality rates. The regional variation in excess death per million persons is shown in Fig. A1 (Online Appendix). We note that the yearly excess mortality figures are very similar even if we use the classical Lee-Carter method^34^ to predict mortality rates (Fig. A2, Online Appendix). The Pearson correlation coefficients between the baseline excess mortality values and the values calculated using the Lee-Carter model for the three years are, 0.96, 0.98 and 0.80, respectively. Perhaps unsurprisingly, if we estimate excess mortality on yearly rather than weekly data, the results remain virtually the same (Fig. A3, Online Appendix). And they do not change significantly even if we use aggregate figures rather than age-specific mortality data (Fig. A4, Online Appendix).

### The connection between regional life expectancy and excess death

**Figure 3:**
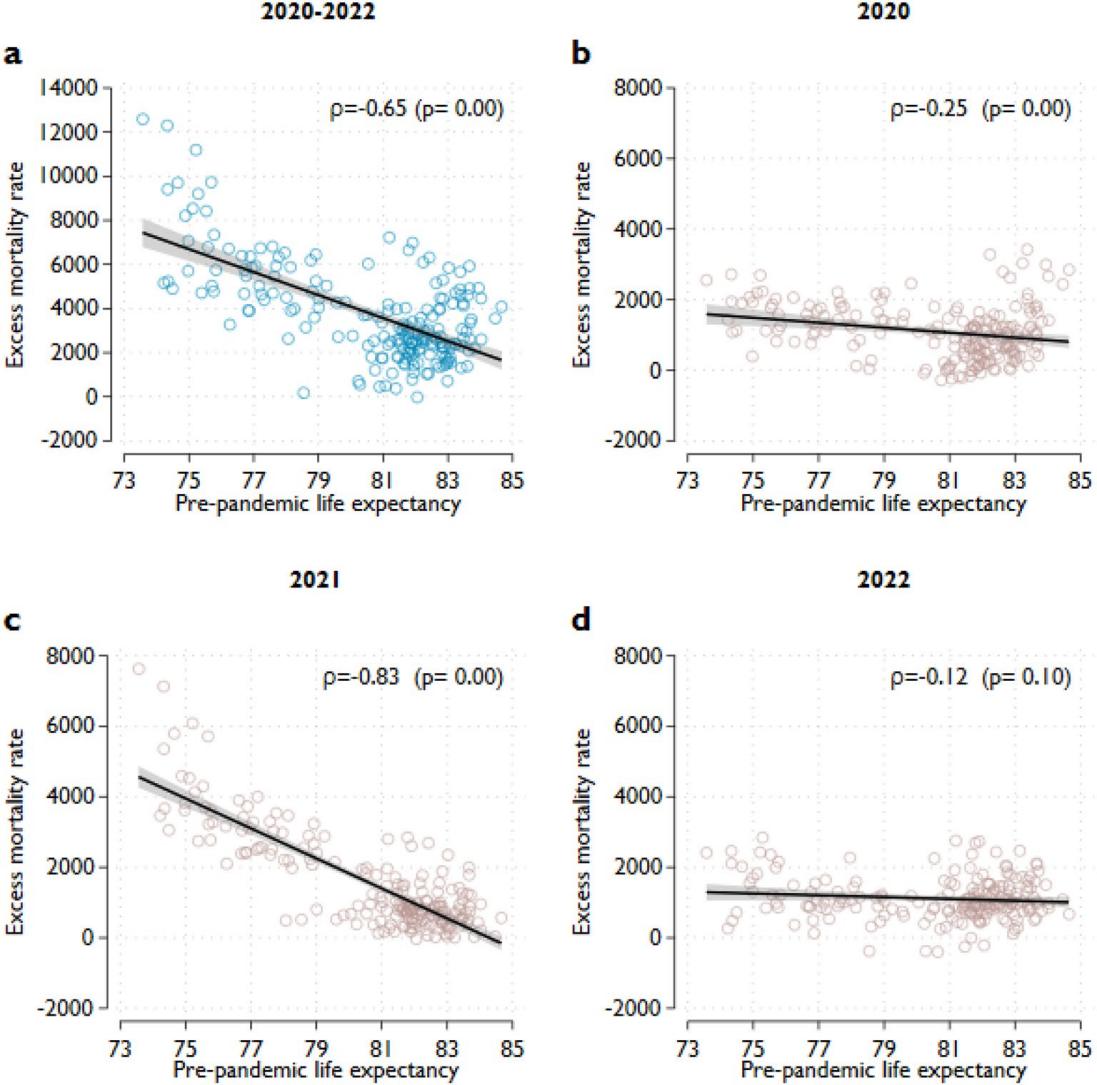
Excess mortality rates as a function of pre-pandemic life expectancy. Notes: Excess mortality rates reflect the difference between the observed and predicted mortality rates. Predicted mortality rates are projected from the observed mortality rates between 2015 and 2019. The projection accounts for seasonality and linear time trends in mortality rates. The excess mortality of the total population is the weighted average of the age-specific excess mortality rates, where the weights are the population shares of the age groups on January 1. Pre-pandemic life expectancy is defined as the average life expectancy over 2015–2019. The lines show the estimated linear relationships between pre-pandemic life expectancy and the excess mortality rate. The shaded areas represent 95% confidence intervals.

There is a clear negative and strong association between pre-pandemic regional life expectancy and excess mortality rate for the whole period, as indicated by a statistically significant and high Pearson correlation coefficient of − 0.65. (Fig. 3a). The regression results further elucidate the implications of this correlation in the context of excess deaths. Specifically, a one-year lower pre-pandemic life expectancy was associated with 521 more excess deaths per million inhabitants (95% CI 411–631) throughout the three years of 2020–2022 (Table 1, Panel A). This impact is considerable given that the average excess death per million population was 3759 across all regions and the three years. This strong association was also indicated by the fact that pre-pandemic life expectancy alone explained 42% of the regional variation in excess deaths for the 2020–2022 period.

**Table 1:** Linear regression analysis of regional excess mortality rates and pre-pandemic life expectancy.

	(1)	(2)	(3)	(4)
	2020	2021	2022	2020–2022
Panel A				
Pre-pandemic life expectancy	− 70.4***	− 425.4***	− 25.6	− 521.3***
	(19.0)	(28.1)	(17.4)	(55.8)
Country FE	No	No	No	No
*R*-squared	0.06	0.69	0.01	0.42
*N*	201	201	201	201
Panel B				
Pre-pandemic life expectancy	62.0	− 133.3***	24.8	− 46.5
	(54.0)	(37.8)	(50.1)	(95.4)
Country FE	Yes	Yes	Yes	Yes
*R*-squared	0.50	0.92	0.63	0.81
*N*	201	201	201	201

Dependent variable: regional excess mortality rate (excess mortality per million population). Excess mortality rates reflect the difference between the observed and predicted mortality rates. Predicted mortality rates are projected from the observed mortality rates between 2015 and 2019. The projection accounts for seasonality and linear time trends in mortality rates. The excess mortality of the total population is the weighted average of the age-specific excess mortality rates, where the weights are the population shares of the age groups on January 1. Pre-pandemic life expectancy is defined as the average life expectancy over 2015–2019. Heteroskedasticity-robust standard errors are in parentheses.

*p < 0.10, **p < 0.05, ***p < 0.01.

While a negative relationship exists for the entire period, different subperiods have distinct patterns. While excess mortality and pre-pandemic life expectancy were slightly negatively correlated in 2020, the correlation shows a very strong association in 2021, with Pearson coefficients of − 0.25 and − 0.85 for the respective years. In 2021, pre-pandemic life expectancy alone explained 69% of the regional variation in excess mortality rates, and the regression results show that a lower one-year pre-pandemic life expectancy was associated with 425 more excess deaths per million persons in this single year (95% CI 370–481). However, as we move into 2022, the relationship between life expectancy and excess deaths disappears. These results are robust to different calculations of the excess mortality rates, such as using a shorter baseline period or quadratic time trends (Fig. A5, Online Appendix), and the results are also unchanged when examining excess mortality rates from the classical Lee-Carter model (Fig. A6, Online Appendix). However, it is worth pointing out that the estimated excess mortality rates could change slightly in some cases if a substantially different baseline period was used^35^. We see similar patterns for males and females (Tables A2 and A3, Online Appendix).

**Figure 4:**
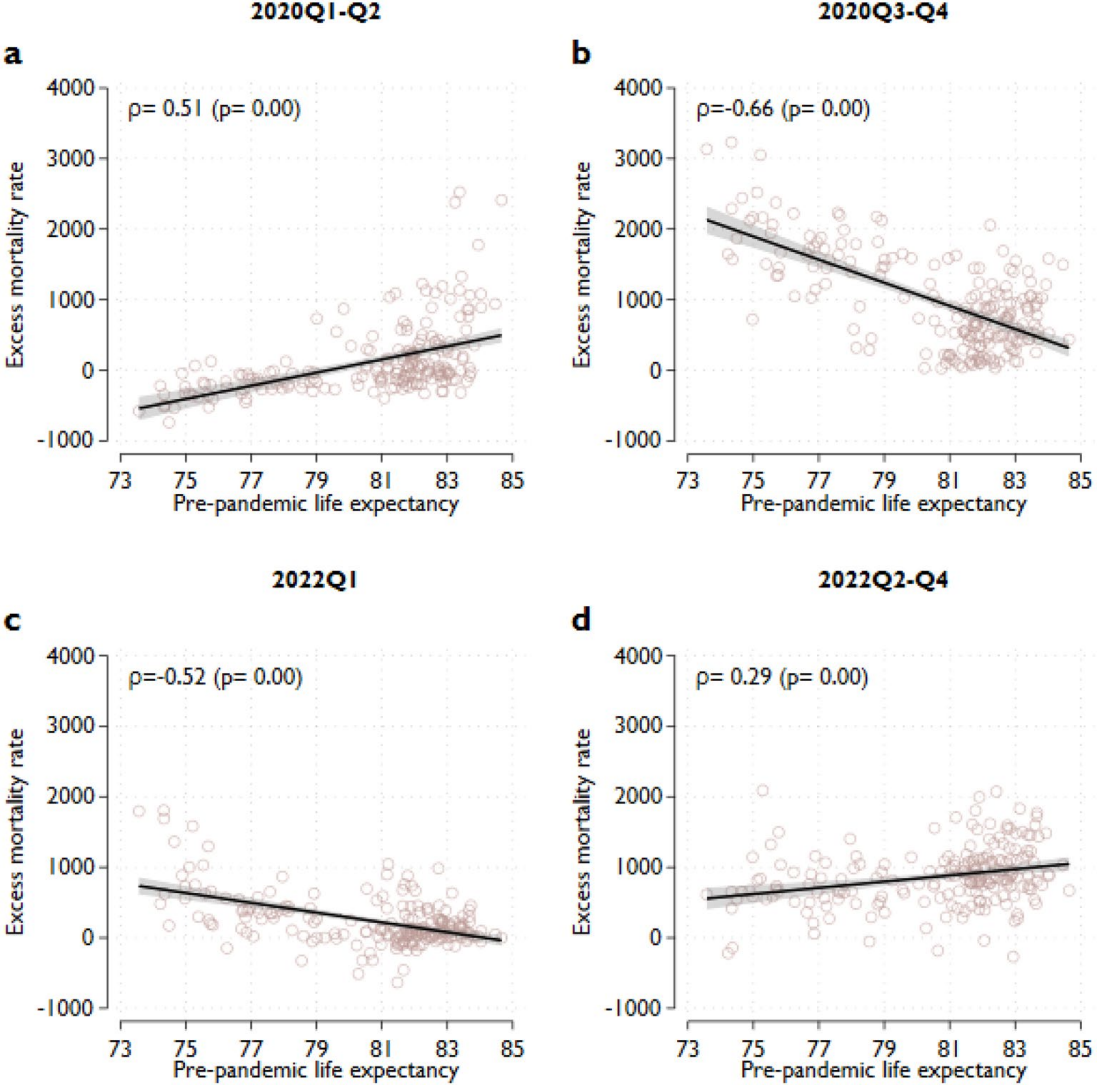
The relationship between excess mortality rates and pre-pandemic life expectancy in different periods of 2020 and 2022. Notes: Excess mortality rates reflect the difference between the observed and predicted mortality rates. Predicted mortality rates are projected from the observed mortality rates between 2015 and 2019. The projection takes accounts for seasonality and linear time trends in mortality rates. The excess mortality of the total population is the weighted average of the age-specific excess mortality rates, where the weights are the population shares of the age groups on January 1. Pre-pandemic life expectancy is defined as the average life expectancy over 2015–2019. The lines show the estimated linear relationships between pre-pandemic life expectancy and the excess mortality rate. The shaded areas represent 95% confidence intervals.

To better understand the relationship between excess mortality rate and pre-pandemic life expectancy, we divided 2020 and 2022 into quarters (Fig. A7, Online Appendix). Looking at the pre-pandemic life expectancy-excess mortality rate relationship by quarter, we observed that the negative correlation only exists between the third quarter of 2020 and the first quarter of 2022. During the first wave of the pandemic (in the first half of 2020), regions with the highest life expectancy also had the highest excess mortality rates, resulting in a significantly positive correlation rate of 0.52. (Fig. 4a). In this period, 1-year higher pre-pandemic life expectancy was associated with 94 more excess deaths per million inhabitants (95% CI 72–115) (Table A1, Online Appendix). A similar positive, though weak association was observed in the second to fourth quarters of 2022, with a correlation coefficient of 0.29 (Fig. 4d). In these three quarters, a higher one-year pre-pandemic life expectancy was associated with 44 more excess deaths per million inhabitants (95% CI 22–66).

Adding country fixed effects to the estimation decreases the parameter of the life expectancy substantially in all periods, reflecting that country dummy variables absorb a substantial fraction of regional variation in pre-pandemic life expectancy. However, the positive parameter in the first part of 2020 and the negative coefficient for 2021 remain significant (Table 1, Panel B).

**Figure 5:**
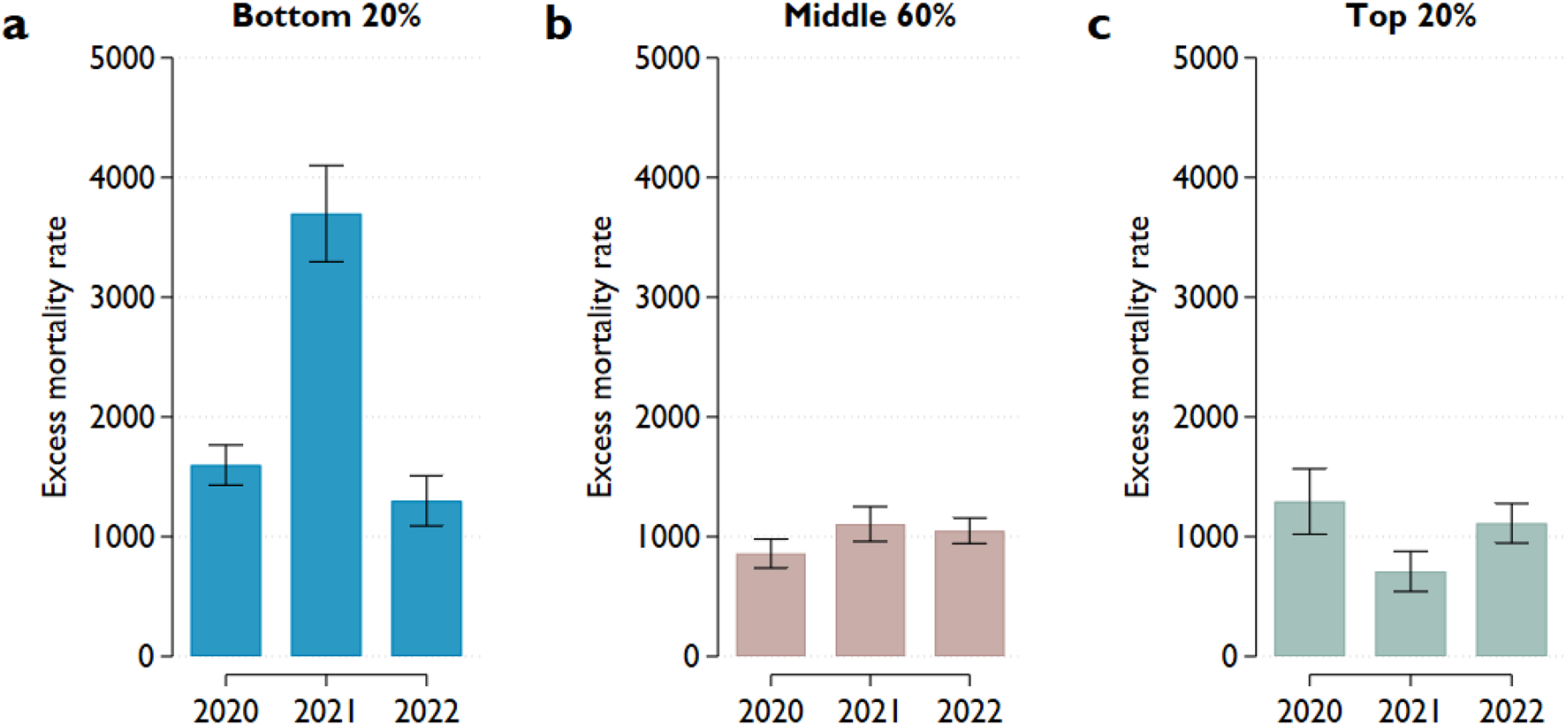
Average excess mortality rates in groups defined by pre-pandemic life expectancy. Notes: Pre-pandemic life expectancy is defined as the average life expectancy over 2015–2019. The bottom (top) 20% includes 20% of the regions with the lowest (highest) pre-pandemic life expectancy. Excess mortality rates reflect the difference between the observed and predicted mortality rates. Predicted mortality rates are projected from the observed mortality rates between 2015 and 2019. The projection accounts for seasonality and linear time trends in mortality rates. The excess mortality of the total population is the weighted average of the age-specific excess mortality rates, where the weights are the population shares of the age groups on January 1. The whiskers represent 95% confidence intervals.

To examine the different time trends in excess mortality rates between regions with lower and higher health capital (pre-pandemic life expectancy), we calculated annual excess mortality rates for three groups: the top 20%, the middle 60% and the bottom 20%, based on pre-pandemic life expectancy (Fig. 5). This stratification revealed that the strong negative connection between the two variables in 2021 (Fig. 3) could be attributed to the dramatic increase in excess mortality in regions with the lowest pre-pandemic life expectancy (the bottom 20%). In contrast, regions with the highest pre-pandemic life expectancy (the top 20%) experienced a slight decline in excess mortality from 2020 to 2021, but an almost similar increase can be observed in 2022. In regions that fall in the middle 60%, the excess mortality rate was remarkably similar in the three subsequent years of the COVID-19 pandemic, with around 1000 per million inhabitants.

### The changing variation in life expectancy

The changing relationship between pre-pandemic life expectancy and excess mortality, especially the strong negative association between them in 2021, raises the issue of changing differences in regional mortality. Since life expectancy at birth describes the annual mortality conditions in a given area, it is suitable for measuring the mortality burden of the pandemic in consecutive years and its impact on the differences in mortality conditions across Europe. In the years before the pandemic, a moderate increase in life expectancy was generally observed in the surveyed regions. Average regional life expectancy slowly improved from 80.3 years in 2015 to 81.0 years in 2019. When the pandemic hit Europe, this indicator fell to 80.3 in 2020 and decreased further to 80.0 in the following years. Our data show a clear rebound in 2022 when average life expectancy almost reached 80.6 years.

**Figure 6:**
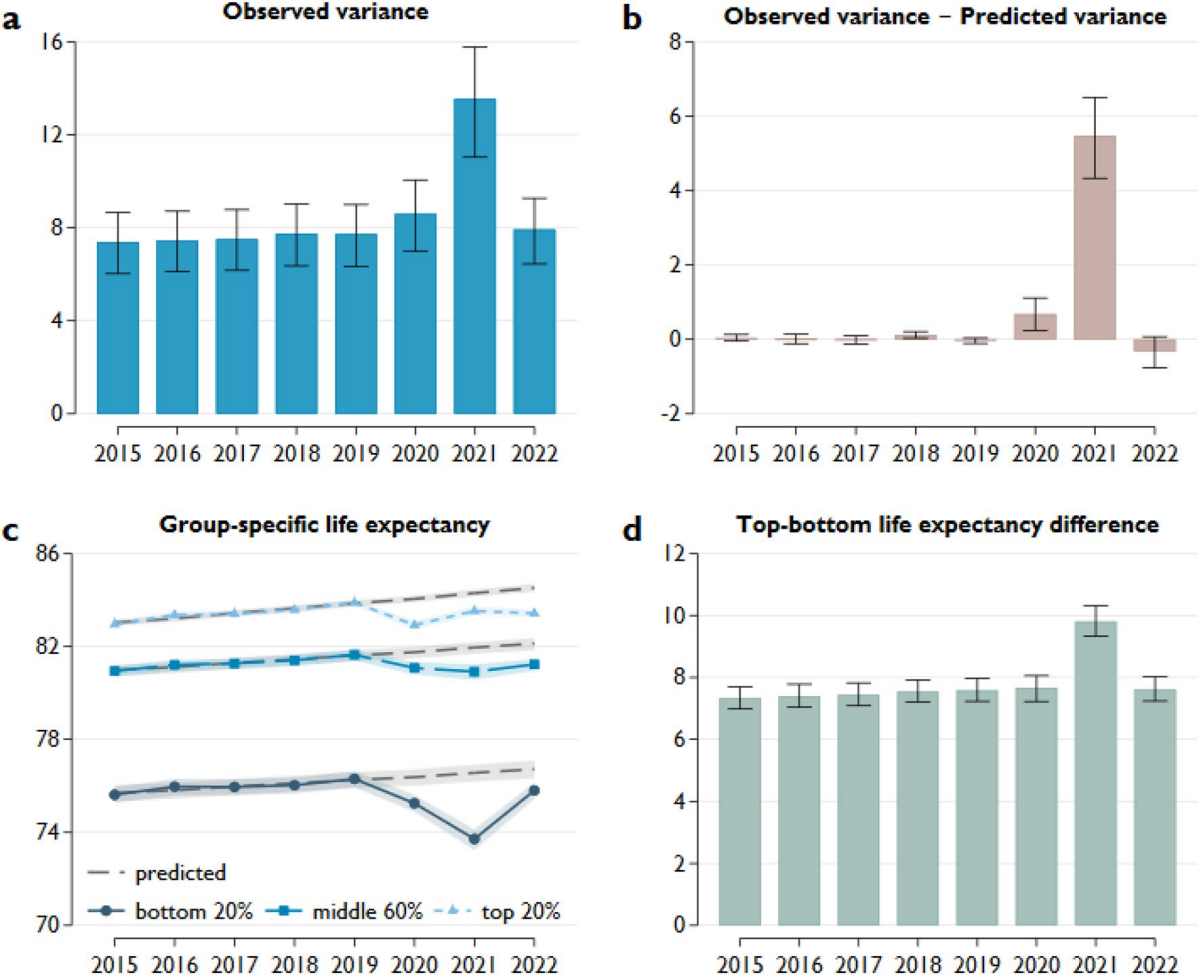
Variance in life expectancy. Notes: (**a**) The variance of life expectancy based on 201 European NUTS 2 regions. (**b**) The difference between the observed and predicted variance, where predicted variance is the variance of regional life expectancy calculated using the predicted mortality rates. (**c**) The bottom (top) 20% includes 20% of the regions with the lowest (highest) pre-pandemic life expectancy, defined as the average life expectancy over 2015–2019. The predicted life expectancy is calculated from the predicted mortality rates. Predicted mortality rates are projected from the observed mortality rates between 2015 and 2019. The projection takes accounts for seasonality and linear time trends in mortality rates. (**d**) The difference between the average life expectancy of the top and bottom 20% of the regions. The whiskers and shaded areas represent 95% confidence intervals calculated from 1000 bootstrap samples.

However, from the perspective of regional differences, the change in the variance of life expectancy is more relevant. This indicator was relatively stable over five years before the pandemic, ranging between 7.4 and 7.8 (Fig. 6a). This stability means that the improvement in life expectancy was a general phenomenon in the continent instead of restricted to some regions, so the variance remained unchanged. The outbreak of the pandemic brought remarkable changes. The variance increased slightly to 8.6 in 2020 (95% CI 7.0–10.1) and jumped to 13.5 in 2021 (95% CI 11.0–15.8) but fell back to 7.9 (95% CI 6.5–9.3), around the pre-crisis level, in 2022. These results suggest that the pandemic significantly increased the regional differences in life expectancy in 2021 and to a smaller extent in 2020. Similar patterns were observed when the mid-year population was used to calculate life expectancy instead of the population on January 1 (Fig. A8, Online Appendix).

The difference between the observed and predicted variance was calculated to identify the contribution of the pandemic to the change in the variance of regional life expectancy. The latter was derived from a projection using mortality rates from 2015 to 2019. This calculation showed that the pandemic increased the variance in life expectancy by 5.5 in 2021 (95% CI 4.3–6.5) (Fig. 6b), corresponding to a 68% increase over the predicted variance. It also means that the regional differences in life expectancy would have stayed around the pre-pandemic level without the emergence of COVID-19. The difference between the observed and predicted variances indicates a significantly small positive impact of the pandemic in 2020, while its effect was effectively zero in 2022.

It is worth breaking down the changes in the regional variance of life expectancy to determine which part of the distribution has undergone a major adjustment. The observed and predicted life expectancies are presented separately for the countries in the top 20%, bottom 20%, and middle 60% according to their position in a rank of the pre-pandemic life expectancy (Fig. 6c). Focusing on 2021, when the variation in regional life expectancy jumped, there are remarkable differences between the three groups. Compared to the predicted value, the countries in the bottom 20% experienced a reduction in life expectancy of 2.84 years (95% CI 2.65–3.04), much larger than the reductions of 0.77 (95% CI 0.66–0.90) and 1.04 year (95% CI 0.91–1.18) for the top 20% and middle 60%, respectively. This finding indicates that the increase in differences arose from the higher mortality of countries initially with lower life expectancy (health capital; Fig. 5a). The overall reduction in life expectancy was much lower in 2020 with a smaller variance. The reduction was 1.12 years in the top and bottom groups and 0.68 years in the middle group. This finding resonates with the experience described above that the COVID-19 excess mortality during the first wave of the pandemic was higher in developed countries, but this had changed by the second half of 2020. The difference between the predicted and observed life expectancy was about the same across the three groups in 2022, varying from 0.90 to 1.09 years. Indeed, observed life expectancy in almost all regions was far below that expected without the pandemic (Fig. A9, Online Appendix).

A similar picture emerges if we focus on the life expectancy gap between the countries with the highest and lowest pre-pandemic life expectancies (Fig. 6d). The slight increase in the so-called top–bottom difference did not exceed the confidence intervals from 2015 to 2019, and this trend continued in the first year of the COVID-19 pandemic. However, a significant jump in this indicator occurred in 2021, followed by a return to the original trend in 2022. The same results were obtained when these investigations were repeated separately for males and females (Fig. A10 and A11, Online Appendix).

**Figure 7:**
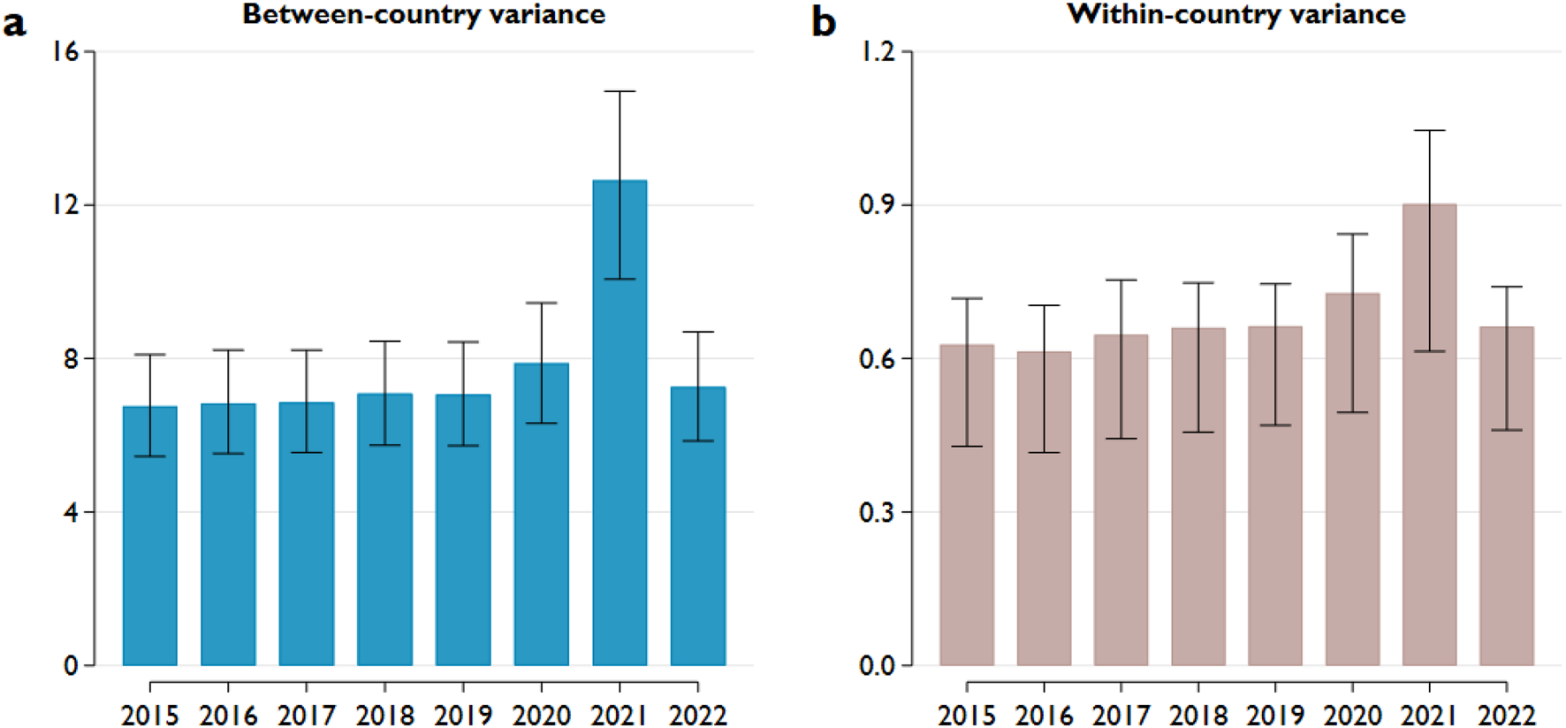
Between-country and within-country variance in life expectancy. Notes: (**a**) Between-country and (**b**) within-country variance in regional life expectancy. The whiskers represent 95% confidence intervals calculated from 1000 bootstrap samples.

As described above, approximately 90% of the variance in regional pre-pandemic life expectancy was due to country-level differences. Therefore, it is very important to determine whether the increase in variance due to COVID-19 was driven exclusively by increasing between-country differences or whether domestic changes also contributed. Consequently, we broke down the variance into between-country and within-country parts to examine this issue (Fig. 7).

Our results indicate that while a large part of the increase in total variance is caused by increasing between-country differences, within-country variation also contributed to it. Moreover, the changes in the within-country differences in consecutive years resemble the patterns observed in the total variance: a slight increase in 2020, a more significant jump in 2021, and a return to pre-pandemic levels in 2022.

## Discussion

The present paper fits into a line of studies^17–20^ examining the mortality burden of COVID-19 across several countries at the subnational level. The first novelty of our research is that we have pursued a comprehensive analysis, including subnational data from 28 European countries, covering three full years of the COVID-19 pandemic, and evaluating the mortality burden of the pandemic through the number of excess mortality and the change in life expectancy too. While most previous research has found significant regional variation, we have taken a step forward by presenting the changing variation in regional mortality across successive phases of the pandemic and exploring the relationship of health capital to this.

Our results demonstrate that while the overall impact of the COVID-19 pandemic was more severe in the regions with lower health capital in Europe, the effects varied widely in spatially and temporally. Over the 3 years of the pandemic, not only did the level of excess mortality rate change, but also its relationship with pre-pandemic life expectancy. The latter is because the time trends in excess mortality rates (and consequently life expectancy) differed considerably between advantaged and disadvantaged regions. The changing variation in regional life expectancy can be interpreted in a broader concept. It is about how societies are resilient to external impacts and adapt to changing circumstances^36,37^.

While life expectancy is an annual indicator, instead of using different years, it is worth dividing the surveyed period into subperiods of different lengths based on the behavior of the pandemic to understand the changing situation. The first period began in the early spring of 2020, when the epidemic reached Europe, and lasted for some months. This phase can be considered an external “random” shock^38,39^. One of the most important factors defining the exposure of a given region to COVID-19 was its embeddedness in international trade and tourism^40,41^. The decision-makers in the various countries had little knowledge of how to fight the new enemy and no time to develop and monitor the different strategies. Therefore, the responses were more or less universal in Europe, and a lockdown was rapidly implemented in most countries^42,43^. Consequently, the pandemic hardly spread to Central and Eastern European countries, where excess mortality rates remained relatively low, mainly because the initial outbreak of the pandemic in Europe occurred in some of the most developed regions that are heavily integrated into the global economy.

The distribution of COVID-19 excess mortality in the first period was mainly driven by the spread of infection. The contributions of all other factors, such as the age structure, the inhabitant’s health status, the level of health services, and the efficiency of state protection, were comparatively lower. An analysis of data from 138 countries during the first wave contagion period found that COVID-19 impacted countries with higher volumes of imports and international tourism more severely^44^. This finding is consistent with our results showing that Western European regions with more intensive trade relations (and generally higher life expectancy) experienced higher excess mortality rates in the first period.

The most significant part of the pandemic in terms of deaths and duration lasted from the third quarter of 2020 to the first quarter of 2022. By this time, the countries were over the first shock, and some months had passed since the pandemic’s outbreak. While the number of uncertainties had hardly decreased, governments had more time than in the first phase to assess the situation, develop an overall strategy, and adapt to the pandemic^45,46^. In this period, lower health capital, measured by pre-pandemic life expectancy, was associated with higher regional excess mortality rates, increasing the variance in observed life expectancy. These results suggest that those factors defining pre-pandemic mortality conditions may have shaped the distribution of COVID-19 excess mortality. Therefore, factors such as health status, lifestyle, nutrition, health infrastructure, and access to healthcare services that shape life expectancy in a given region in “normal” times (i.e., health capital) may also have a crucial role in vulnerability to a pandemic.

After the first (almost random) shock, the fundamental patterns of the health condition of societies came to the fore and had a major impact on their performance in the fight against COVID-19. This finding has a significant policy implication since it reveals that those regions where the mortality condition is generally worse need the most external help and support in the future due to their low resilience against an epidemic. In the longer term, various measures should be taken to increase health capital in these areas, which will reduce exposure to external shocks.

The third period started in the first quarter of 2022 when the negative association between pre-pandemic life expectancy and regional excess mortality changed. While the main forces shaping the new pattern of COVID-19 excess mortality remain unclear, it is worth emphasizing that these changes coincided with the moderation of the pandemic. While there were countries where excess mortality still spiked for a while afterward, the overall intensity of the pandemic declined significantly. With the emergence of the less severe Omicron variant of SARS-CoV-2^47,48^, the monthly excess mortality fell by a third in the rest of the year compared to the first quarter of 2022, mainly driven by moderation in the regions in the bottom 20% of pre-pandemic life expectancy. This shift is also reflected in the return of life expectancy variation to its pre-pandemic level. However, further analysis is needed to explain the altered association in the second-fourth quarter of 2022 and the persisting excess death rates. The indirect mortality effects of the pandemic, such as delayed diagnoses of cancer and other non-COVID-19 conditions, may worsen mortality on a longer term horizon^49,50^.

Another potential explanation for the considerable improvement in mortality in the regions in the bottom 20% (i.e., the changing pattern in excess mortality rate) is the harvesting effect^5^, which refers to mortality displacement, where COVID-19 advances deaths that would have occurred within a short period regardless. In addition, the declining contribution of COVID-19 to excess mortality may lead to the increasing role of other “traditional” causes, such as heatwaves^51^ and flu epidemics^52^, in defining the distribution of mortality conditions in Europe.

## Conclusions

Our research aimed to investigate how the COVID-19 pandemic changed the differences between the mortality conditions of the European regions. We first estimated the regional excess mortality rate from the first week of 2020 to the final week of 2022 for 201 NUTS 2 regions in Europe. The results show significant differences in successive years of the pandemic, not only in the size of the mortality burden but also in its geographical distribution. Next, we took pre-pandemic life expectancy as a measure of health capital and compared it with the regional excess mortality rate during the pandemic. We found a significant negative relationship for the whole period, meaning that lower health capital was associated with higher excess mortality per million population. However, we showed that the negative relationship for the entire period masks different processes in the different subperiods. For a few months after the emergence of COVID-19, the relationship was positive, which can be explained by embeddedness in international trade and tourism being the main driver of the epidemic’s spread. The relationship between pre-pandemic life expectancy and excess mortality rates was negative from the third quarter of 2020 to the first quarter of 2022 before becoming slightly positive.

This pattern is consistent with our main results on the changing regional variation of the mortality conditions due to the pandemic. We found that the regional variation in life expectancy that was stable over the 5 years before the pandemic increased slightly in 2020, significantly jumped in 2021, and returned to almost its pre-pandemic level in 2022. The comparison of the predicted life expectancy projected from the mortality rates between 2015 and 2019 and the observed life expectancy confirms that the emergence of the pandemic greatly increased the variation in life expectancy in 2021. This impact was relatively low in 2020 but not significantly different from zero in 2022. By analyzing different parts of the distribution from the perspective of pre-pandemic life expectancy, we found that the increase in variance was due to higher mortality in countries with a lower pre-pandemic life expectancy. In addition, we found that while a large part of the increase in total variance in 2021 was due to increasing between-country differences, within-country variation also contributed to it. While the variance in life expectancy had returned to its pre-pandemic level in 2022, the level of observed life expectancy is far below that expected without the pandemic in almost all regions, indicating that the mortality burdens have not disappeared even in the third pandemic year.

The lesson to be learned from our results is that regions with lower health capital face greater risks in case of future global health shocks. The greater vulnerability of areas with lower life expectancy increases the inequality in health conditions. If the shock permanently reduces life expectancy, inequalities may continue to increase.

### Supplementary Information

Supplementary Information.

## Data Availability

The results of the study are based on publicly available Eurostat datasets (demo_r_mwk2_05, demo_r_d2jan). They were downloaded on 8 June 2023. See 10.6084/m9.figshare.25211888 for the code necessary for replication of the results.
