# Supplementary material for: Inequalities in regional excess mortality and life expectancy during the COVID-19 pandemic in Europe
Source: Sci Rep. 2024 Feb 15;14:3835. doi: 10.1038/s41598-024-54366-5 (PMC10869827; doi:10.1038/s41598-024-54366-5)
Supplement: Supplementary file 1 — Supplementary Information. [file 41598_2024_54366_MOESM1_ESM.docx]

# Online Appendix

Fig. A1: Excess mortality rates by year


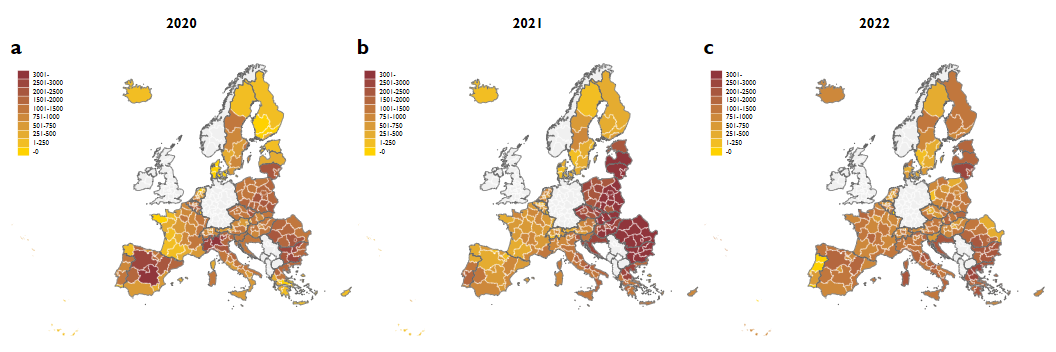


Notes: Excess mortality rates reflect difference between the observed and predicted mortality rates. Predicted mortality rates are projected from the observed mortality rates between 2015 and 2019. The projection accounts for seasonality and linear time trends in mortality rates. The excess mortality of the total population is the weighted average of the age-specific excess mortality rates where the weights are the population shares of the age groups on January 1.

Fig. A2: Excess mortality rates, baseline values and the results of a Lee-Carter model


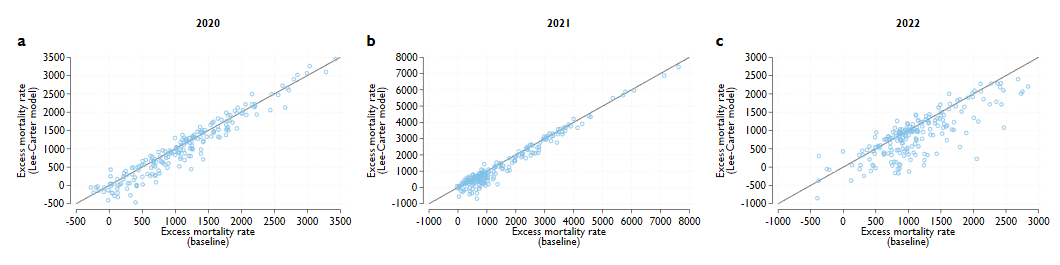


Notes: Excess mortality rates reflect difference between the observed and predicted mortality rates. Predicted mortality rates are projected from the observed mortality rates between 2015 and 2019. In the baseline specification, the projection accounts for seasonality and linear time trends in mortality rates. As an alternative specification, a classical Lee-Carter model was used to calculate excess mortality rates. The excess mortality of the total population is the weighted average of the age-specific excess mortality rates where the weights are the population shares of the age groups on January 1. The line indicates the 45-degree line.

Fig. A3: Excess mortality rates, baseline values and the results from yearly data


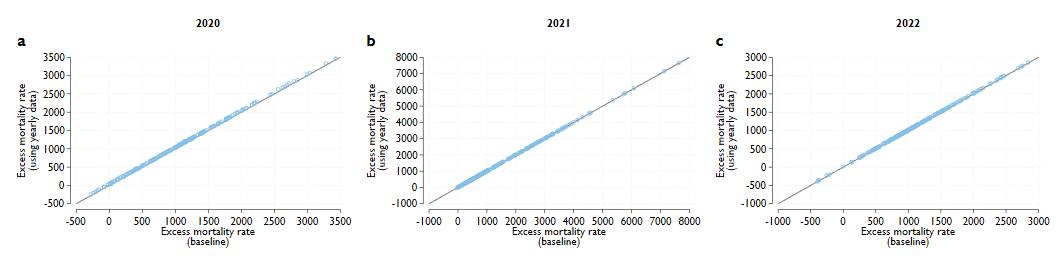


Notes: Excess mortality rates reflect difference between the observed and predicted mortality rates. Predicted mortality rates are projected from the observed mortality rates between 2015 and 2019. In the baseline specification, the projection accounts for seasonality and linear time trends in mortality rates. As an alternative specification, yearly data was used to calculate excess mortality rates. The excess mortality of the total population is the weighted average of the age-specific excess mortality rates where the weights are the population shares of the age groups on January 1. The line indicates the 45-degree line.

Fig. A4: Excess mortality rates, baseline values and the results using all ages combined


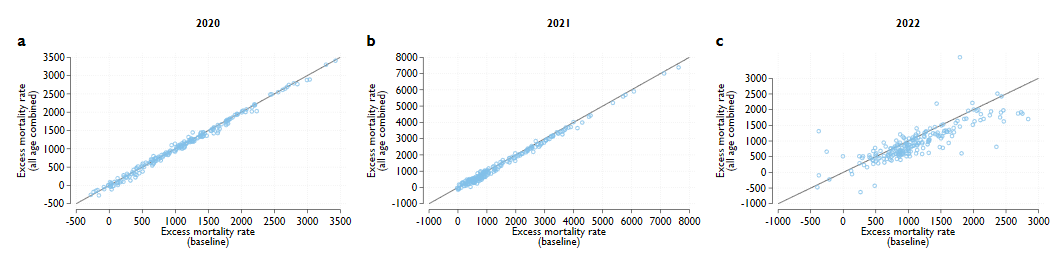


Notes: Excess mortality rates reflect difference between the observed and predicted mortality rates. Predicted mortality rates are projected from the observed mortality rates between 2015 and 2019. In the baseline specification, the projection accounts for seasonality and linear time trends in mortality rates. As an alternative specification, all age categories were combined and the total mortality values were used to calculate excess mortality rates. The excess mortality of the total population is the weighted average of the age-specific excess mortality rates where the weights are the population shares of the age groups on January 1. The line indicates the 45-degree line.

Fig. A5: Sensitivity of the relationship between excess mortality rates and pre-pandemic life expectancy


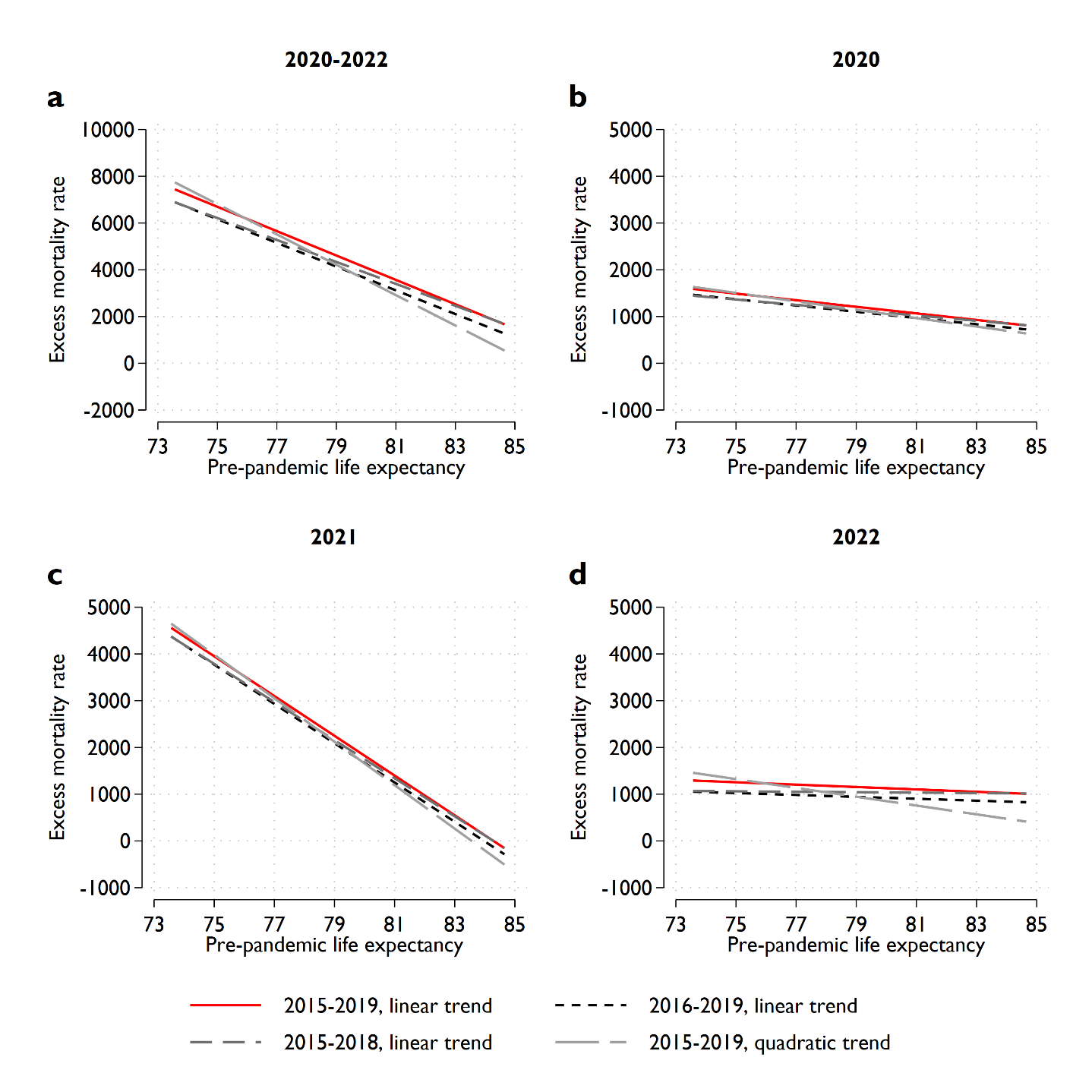


Notes: Excess mortality rates reflect the difference between the observed and predicted mortality rates. Predicted mortality rates are projected using different models. The baseline model uses the years 2015-2019 and accounts for seasonality and linear time trends in mortality rates (red line). The alternative models use different time periods and/or different time trends (gray lines). The excess mortality of the total population is the weighted average of the age-specific excess mortality rates where the weights are the population shares of the age groups on January 1. Pre-pandemic life expectancy is defined as the average life expectancy over 2015–2019. The lines show the estimated linear relationships between pre-pandemic life expectancy and the excess mortality rate.

Fig. A6: The relationship between excess mortality rates and pre-pandemic life expectancy – results using the Lee-Carter model


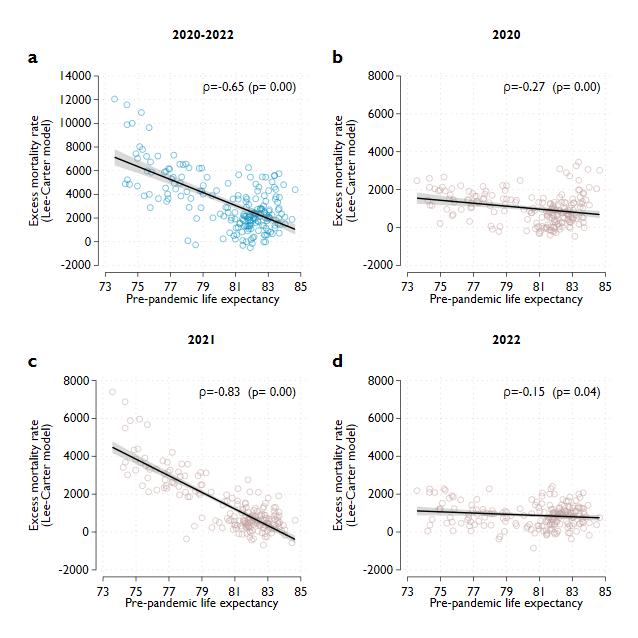


Notes: Excess mortality rates reflect the difference between the observed and predicted mortality rates. Predicted mortality rates are projected from the observed mortality rates between 2015 and 2019. The projection based on the classical Lee-Carter model. The excess mortality of the total population is the weighted average of the age-specific excess mortality rates, where the weights are the population shares of the age groups on January 1. Pre-pandemic life expectancy is defined as the average life expectancy over 2015–2019. The lines show the estimated linear relationships between pre-pandemic life expectancy and the excess mortality rate. The shaded areas represent 95% confidence intervals.

Fig. A7: The relationship between excess mortality rates and pre-pandemic life expectancy in quarters of 2020 and 2022


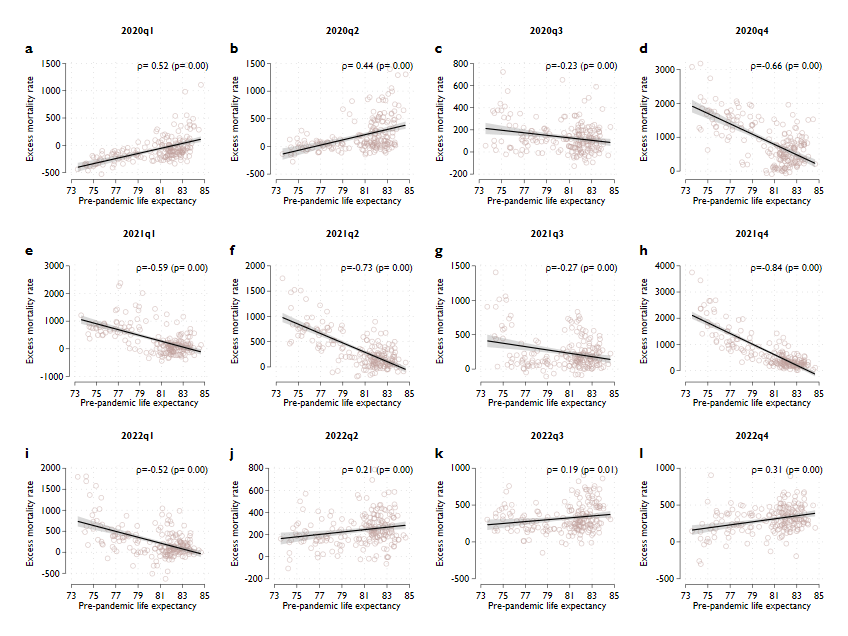


Notes: Excess mortality rates reflect difference between the observed and predicted mortality rates. Predicted mortality rates are projected from the observed mortality rates between 2015 and 2019. The projection accounts for seasonality and linear time trends in mortality rates. The excess mortality of the total population is the weighted average of the age-specific excess mortality rates where the weights are the population shares of the age groups on January 1. Pre-pandemic life expectancy is defined as the average life expectancy over 2015–2019. The lines show the estimated linear relationships between pre-pandemic life expectancy and the excess mortality rate. The shaded areas represent 95% confidence intervals.

Fig. A8: Sensitivity of the variance of life expectancy


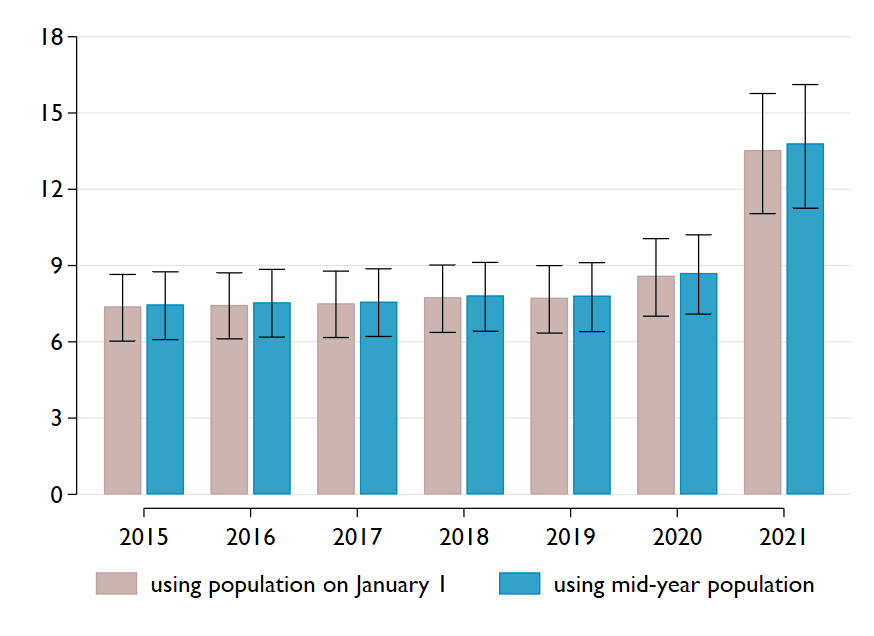


Notes: The brown bars show the variance of life expectancy when life expectancy is calculated using population on January 1. The blue bars show the variance of life expectancy when life expectancy is calculated using the mid-year population. Confidence intervals are calculated from 1000 bootstrap samples. Whiskers represent 95% confidence intervals.

Fig. A9: The difference between the observed and predicted life expectancy by region


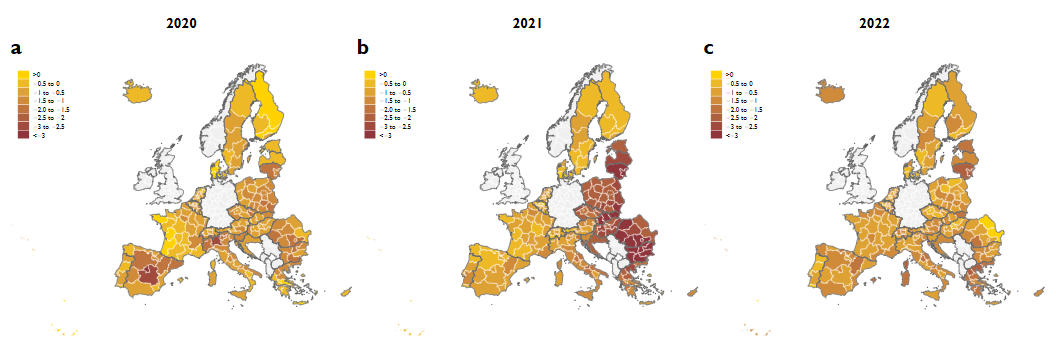


Notes: Life expectancy at birth. The predicted life expectancy is calculated from the predicted mortality rates. Predicted mortality rates are projected from the observed mortality rates between 2015 and 2019. The projection accounts for seasonality and linear time trends in mortality rates.

Fig. A10: Variance of life expectancy and trends for groups defined by pre-pandemic life expectancy, females


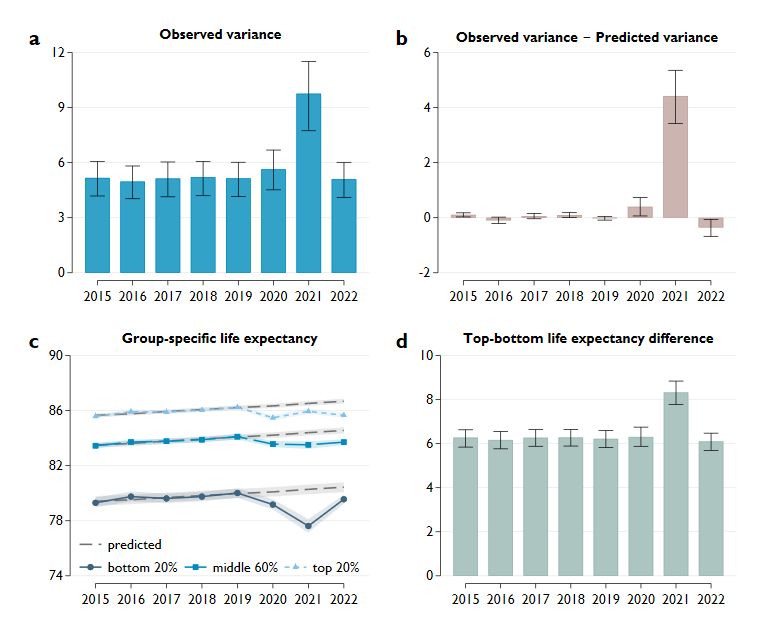


Notes: (**a**) The variance of life expectancy based on 201 European NUTS 2 regions. (**b**) The difference between the observed and predicted variance, where predicted variance is the variance of regional life expectancy calculated using the predicted mortality rates. (**c**) The bottom (top) 20% includes 20% of the regions with the lowest (highest) pre-pandemic life expectancy, defined as the average life expectancy over 2015–2019. The predicted life expectancy is calculated from the predicted mortality rates. Predicted mortality rates are projected from the observed mortality rates between 2015 and 2019. The projection accounts for seasonality and linear time trends in mortality rates. (**d**) The difference between the average life expectancy of the top and bottom 20% of the regions. The whiskers and shaded areas represent 95% confidence intervals calculated from 1000 bootstrap samples.

Fig. A11: Variance of life expectancy and trends for groups defined by pre-pandemic life expectancy, males


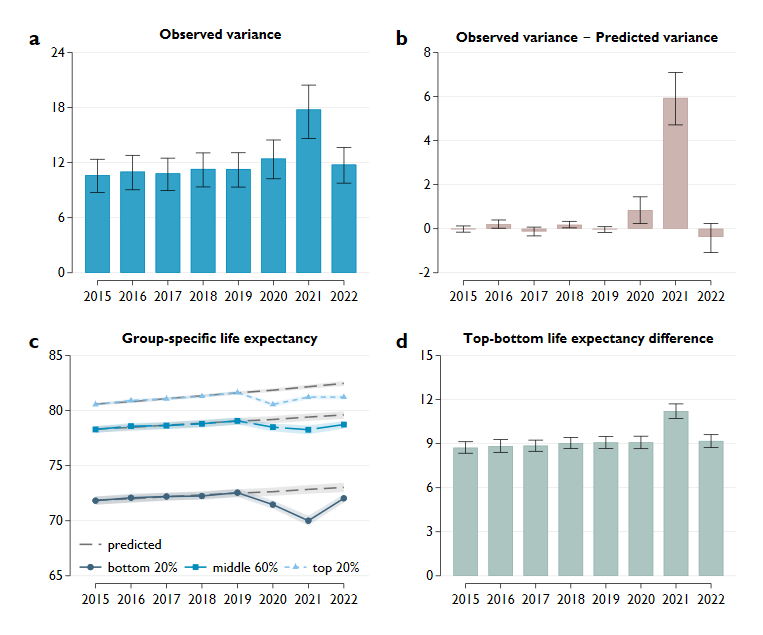


Notes: (**a**) The variance of life expectancy based on 201 European NUTS 2 regions. (**b**) The difference between the observed and predicted variance, where predicted variance is the variance of regional life expectancy calculated using the predicted mortality rates. (**c**) The bottom (top) 20% includes 20% of the regions with the lowest (highest) pre-pandemic life expectancy, defined as the average life expectancy over 2015–2019. The predicted life expectancy is calculated from the predicted mortality rates. Predicted mortality rates are projected from the observed mortality rates between 2015 and 2019. The projection accounts for seasonality and linear time trends in mortality rates. (**d**) The difference between the average life expectancy of the top and bottom 20% of the regions. The whiskers and shaded areas represent 95% confidence intervals calculated from 1000 bootstrap samples.

Table A1: The relationship between excess mortality rates and pre-pandemic life expectancy in different periods of 2020 and 2022

| Panel A | | | | |
| --- | --- | --- | --- | --- |
|  | (1) | (2) | (3) | (4) |
|  | 2020Q1-Q3 | 2020Q4 | 2022Q1 | 2022Q2-Q4 |
| Pre-pandemic life expectancy | 93.5^***^ (10.7) | −163.9^***^ (14.3) | −69.6^***^ (11.3) | 44.1^***^ (11.0) |
| Country FE | No | No | No | No |
| R-squared | 0.26 | 0.44 | 0.27 | 0.08 |
| N | 201 | 201 | 201 | 201 |
| Panel B | | | | |
|  | (1) | (2) | (3) | (4) |
|  | 2020Q1-Q3 | 2020Q4 | 2022Q1 | 2022Q2-Q4 |
| Pre-pandemic life expectancy | 112.3^***^ (37.5) | −50.3 (32.7) | −11.5 (20.6) | 36.3 (36.0) |
| Country FE | Yes | Yes | Yes | Yes |
| R-squared | 0.47 | 0.77 | 0.84 | 0.56 |
| N | 201 | 201 | 201 | 201 |

Notes: Dependent variable: regional excess mortality rate (excess mortality per million population). Excess mortality rates reflect the difference between the observed and predicted mortality rates. Predicted mortality rates are projected from the observed mortality rates between 2015 and 2019. The projection accounts for seasonality and linear time trends in mortality rates. The excess mortality of the total population is the weighted average of the age-specific excess mortality rates where the weights are the population shares of the age groups on January 1. Pre-pandemic life expectancy is defined as the average life expectancy of 2015–2019. Heteroskedasticity-robust standard errors are in parentheses. * p<0.10, ** p<0.05, *** p<0.01

Table A2: The relationship between excess mortality rates and pre-pandemic life expectancy, females

| Panel A | | | | |
| --- | --- | --- | --- | --- |
|  | (1) | (2) | (3) | (4) |
|  | 2020 | 2021 | 2022 | 2020-2022 |
| Pre-pandemic life expectancy | −40.3^*^ (21.8) | −474.5^***^ (33.6) | −16.8 (19.3) | −531.6^***^ (62.2) |
| Country FE | No | No | No | No |
| R-squared | 0.02 | 0.62 | 0.00 | 0.32 |
| N | 201 | 201 | 201 | 201 |
| Panel B | | | | |
|  | (1) | (2) | (3) | (4) |
|  | 2020 | 2021 | 2022 | 2020-2022 |
| Pre-pandemic life expectancy | 77.8 (62.5) | −128.8^***^ (47.8) | 44.9 (54.9) | −6.1 (106.7) |
| Country FE | Yes | Yes | Yes | Yes |
| R-squared | 0.45 | 0.90 | 0.53 | 0.76 |
| N | 201 | 201 | 201 | 201 |

Notes: Dependent variable: regional excess mortality rate (excess mortality per million population). Excess mortality rates reflect the difference between the observed and predicted mortality rates. Predicted mortality rates are projected from the observed mortality rates between 2015 and 2019. The projection accounts for seasonality and linear time trends in mortality rates. The excess mortality of the total population is the weighted average of the age-specific excess mortality rates where the weights are the population shares of the age groups on January 1. Pre-pandemic life expectancy is defined as the average life expectancy over 2015 The projection account for seasonality and linear time trends in mortality rates 2019. Heteroskedasticity-robust standard errors are in parentheses. * p<0.10, ** p<0.05, *** p<0.01

Table A3: The relationship between excess mortality rates and pre-pandemic life expectancy, males

| Panel A | | | | |
| --- | --- | --- | --- | --- |
|  | (1) | (2) | (3) | (4) |
|  | 2020 | 2021 | 2022 | 2020-2022 |
| Pre-pandemic life expectancy | −82.2^***^ (18.8) | −361.7^***^ (26.0) | −24.4 (17.1) | −468.3^***^ (54.1) |
| Country FE | No | No | No | No |
| R-squared | 0.10 | 0.65 | 0.01 | 0.39 |
| N | 201 | 201 | 201 | 201 |
| Panel B | | | | |
|  | (1) | (2) | (3) | (4) |
|  | 2020 | 2021 | 2022 | 2020-2022 |
| Pre-pandemic life expectancy | 67.0 (50.3) | −98.6^**^ (39.2) | 52.4 (51.5) | 20.9 (98.9) |
| Country FE | Yes | Yes | Yes | Yes |
| R-squared | 0.53 | 0.90 | 0.61 | 0.78 |
| N | 201 | 201 | 201 | 201 |

Notes: Dependent variable: regional excess mortality rate (excess mortality per million population). Excess mortality rates reflect the difference between the observed and predicted mortality rates. Predicted mortality rates are projected from the observed mortality rates between 2015 and 2019. The projection accounts for seasonality and linear time trends in mortality rates. The excess mortality of the total population is the weighted average of the age-specific excess mortality rates where the weights are the population shares of the age groups on 1 January. Pre-pandemic life expectancy is defined as the average life expectancy over 2015 The projection account for seasonality and linear time trends in mortality rates 2019. Heteroskedasticity-robust standard errors are in parentheses. * p<0.10, ** p<0.05, *** p<0.01
